# B Cells in Rheumatoid Arthritis: From Pathogenic Players to Disease Biomarkers

**DOI:** 10.1155/2014/681678

**Published:** 2014-04-29

**Authors:** Serena Bugatti, Barbara Vitolo, Roberto Caporali, Carlomaurizio Montecucco, Antonio Manzo

**Affiliations:** Rheumatology and Translational Immunology Research Laboratories (LaRIT), Division of Rheumatology, IRCCS Policlinico San Matteo Foundation/University of Pavia, Piazzale Golgi 2, 27100 Pavia, Italy

## Abstract

The therapeutic benefit of depleting B cells in rheumatoid arthritis (RA) has refocused attention on B cells with increasing awareness on their role in autoimmunity and their function beyond autoantibody production. The rapid increase in our comprehension of B-cell pathobiology is progressively opening novel perspectives in the area of B cell-targeted therapies with the expectation to define more specific approaches able to preserve the homeostasis of the humoral response while disrupting the pathogenic components. In parallel, B-cell activity in RA is starting to be explored in its clinical value, in search of novel biomarkers embedded in the pathogenic process that could help classifying the disease and predicting its heterogeneous outcome beyond inflammation dynamics. In this review, we summarize current knowledge on the multiple roles that B cells play in several aspects of RA. We also analyze their distribution and potential function in different anatomic compartments with specific reference to the main sites in which the disease may be sustained and exert its detrimental effects: the systemic circulation, synovium, bone marrow, and draining lymph nodes. We also highlight novel data encouraging further research in the field of biomarkers related to B cells and their regulatory factors.

## 1. Introduction

The history of the pathogenic involvement of B cells in rheumatoid arthritis (RA) has spanned glories and hurdles. The discovery of rheumatoid factors (RFs) by Waaler in 1937–1939 and Rose in 1948 fueled the attractive hypothesis that RA pathogenesis mostly relied on antigen-antibody reactions in the joints, activating the cascade of complement and promoting chemotactic migration of polymorphs, the final effectors of articular damage [1]. The lack of specificity of RFs for RA rapidly shifted the attention to alternative players, such as macrophages and T cells, which have dominated the scene for decades leading to the development of effective targeted therapies [2]. After years of impasse, the therapeutic benefit and safety of depleting B cells in mice and humans [3, 4] have refocused attention on B cells and their role in autoimmunity beyond autoantibody production [5, 6]. As knowledge on B-cell biopathology increases, developments in the area of B cell-targeted therapies are moving fast [7]. Equally exciting, the cellular and molecular signatures of B-cell activity in patients with RA are starting to be explored in their clinical value, in search of novel biomarkers beyond conventional autoantibodies that could help better classifying the disease and predicting its heterogeneous outcome. In this review, we summarize current knowledge on the multiple and unexpected roles that B cells play in several aspects of RA immunopathology, analyze their redistribution in different anatomic compartments, and highlight novel data encouraging further research in the field of B-cell biomarkers.

## 2. Primary Defects in the Generation of the B-Cell Repertoire and Peripheral Tolerance Checkpoints 

In healthy individuals, most autoreactive B cells are removed at 2 discrete steps [8, 9]. A central B-cell tolerance checkpoint in the bone marrow between early immature and immature B cells removes the vast majority of B cell clones expressing polyreactive antibodies and antinuclear antibodies. A peripheral B-cell tolerance checkpoint further counter selects autoreactive new emigrant/transitional B cells before they enter the long-lived mature naive B cell pool. Central B-cell tolerance is mostly controlled by intrinsic B-cell factors regulating B-cell receptor (BCR) and Toll-like receptor (TLR) signaling as well as the expression levels of the enzyme activation-induced cytidine deaminase (AID), which is required for class-switch recombination and somatic hypermutation of the immunoglobulin (Ig) genes [10]. In contrast, peripheral B-cell tolerance seems to involve extrinsic B-cell factors such as regulatory T cells (Treg) and serum B-cell activating factor (BAFF) concentrations [10].

Both central and peripheral B-cell tolerance checkpoints are defective in RA, resulting in the accumulation of a large number of autoreactive B cells in the mature naive B cell compartment [9]. In untreated patients with active RA, the frequency of polyreactive new emigrant/transitional B cells in the peripheral blood was found to increase for 3.4-fold compared to control subjects, highlighting the inability to remove polyreactive B cells in the bone marrow [9]. Many susceptibility genes associated with RA, such as tyrosine phosphatase nonreceptor type 22 (PTPN22), have been shown to affect BCR signaling pathways. Accordingly, similar central B-cell tolerance defects are observed in healthy single PTPN22 risk allele carriers and in active RA [10]. Increased frequencies of polyreactive new emigrant/transitional B cells indicative of a defective central B-cell tolerance checkpoint are also observed in association with genetic defects of involving TLR signaling and AID activity [10], but the possible association of these susceptibility genes with RA development is currently unknown. Importantly, impaired central B-cell tolerance in patients with RA is not resolved by effective treatment regimens that reduce inflammation, confirming the relevance of intrinsic genetic predispositions over and above the imbalance of proinflammatory cytokines at this early checkpoint [11].

The increased frequency of mature naive B cells expressing both polyreactive and HEp-2-reactive antibodies in patients with RA indicates further defects in peripheral B-cell tolerance checkpoints [9]. Treg function is impaired in RA with respect to suppression of CD4 T cells [12]. In contrast, it has been recently shown that defective regulation of autoreactive B cells in patients with RA is due not only to intrinsic defects in Tregs but also as a result of B-cell resistance to suppression due to resistance to Fas-mediated apoptosis [13]. Increased levels of BAFF, as detected systemically [14, 15] and, more importantly, within the joint compartment of patients with RA [16, 17], may further foster the survival and maturation of autoreactive B cells. The potential sources of BAFF in autoimmune diseases are incredibly diverse and include haematopoietic and nonhaematopoietic cell lineages [18]. Relevantly, BAFF expression is increased in the presence of several types of cytokines and chemokines as well as by the activation of TLRs [18]. The production of BAFF by nonhaematopoietic cells might provide local niches to modulate the survival and function of B cells. Accordingly, BAFF expression by synovial fibroblasts in response to TLR signaling has been shown to promote Ig class switching via AID induction [19, 20]. Furthermore, TLR signals per se are costimulatory and critically regulate T-independent autoreactive B cell activation and isotype-switch of Ig genes outside germinal centres (GCs) [21].

## 3. Different Roles of B Cells in RA Pathobiology 

**Figure 1:**
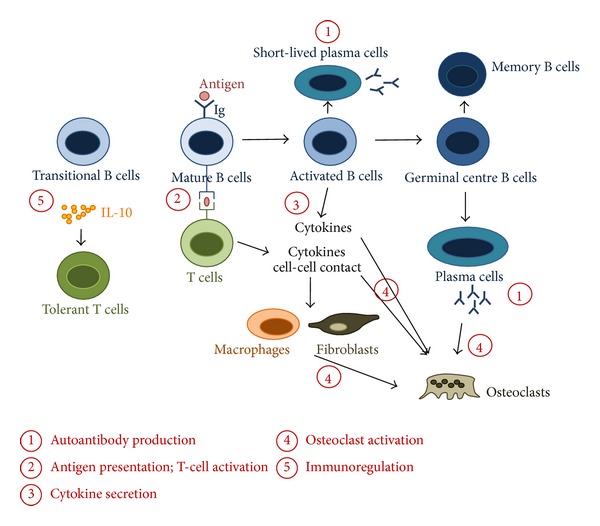
The multiple roles of B cells in rheumatoid arthritis. Immune responses potentially orchestrated by B cells in rheumatoid arthritis. B-T cell interactions result in the activation and differentiation of plasma cells, responsible for the production of autoantibodies (*①*). In turn, activated B cells provide help to T cells and induce differentiation of effector T cells that produce proinflammatory cytokines (*②*). B cells can also impact on other immune and nonimmune cell functions through secretion of cytokines, such as interleukin (IL)-1, IL-6, tumor necrosis factor (TNF)-*α*, and IL-17A (*③*). Proinflammatory cytokines and receptor activator of nuclear factor *κB* ligand (RANKL) produced by activated B cells, T cells, macrophages, and synovial fibroblasts promote the differentiation and activation of osteoclasts, leading to bone resorption (*④*). Further participation of B cells in bone homeostasis is suggested by the recognition that autoantibodies recognizing citrullinated vimentin are able to promote the differentiation of mononuclear cells to osteoclasts (*④*). B cells can also be immunoregulatory through the provision of IL-10 and other mechanisms yet to be elucidated (*⑤*).

The pathogenic roles of circulating and tissue-localized B cells in RA can occur through several mechanisms that include autoantibody production, T-cell activation, and cytokine synthesis. Recent seminal works have also shed new light onto B-cell regulatory functions in the context of immune-mediated inflammation and revealed an unexpected role of B cells in bone homeostasis (Figure 1).

### 3.1. Autoantibody Production

A large number of different antigens are recognized by the antibody repertoire in RA, and the list of RA-associated autoantibodies continues to expand (Table 1). The two most widely studied autoantibody systems, whose importance is also underscored by their inclusion in the clinical management of patients with RA, are RFs and anticitrullinated protein antibodies (ACPA).

Understanding how these autoantibodies are generated has catalyzed considerable efforts in search of the mechanisms that initiate and maintain autoimmunity in RA.

An intriguing line of research suggests that autoreactive responses against self-IgG (generating RFs, which are predominantly nonswitched IgM) may occur outside GCs at least initially in a T-independent fashion [22]. Extrafollicular responses are critically regulated by TLRs and involve self-antigens containing a TLR-ligand that is recognized by a B cell-intrinsic TLR, as is the case of self-IgG [21]. Somatic hypermutation and isotype switch of Ig genes, which classically characterize T cell-dependent GC responses, can also occur extrafollicularly upon TLR signaling, and some RF clones from RA patients are indeed somatically mutated compared to healthy subjects [23]. Importantly, although T cells are not required for the initiation of extrafollicular responses, they substantially amplify and sustain chronic autoantibody production via CD40L and interleukin (IL)-21 signaling [21]. In contrast, immunity to citrullinated antigens is thought to require autoreactive T cells and to develop within classical GC reactions. ACPA are indeed strongly associated with HLA DR alleles [24] and are switched IgG with strong bias toward amino acid replacement mutations [25]. It is worth noting that such postulated disparity in the involvement of TLRs and T cells for the generation of RF and ACPA responses might in part explain the differences in treatment-related changes observed between the two autoantibody systems [26, 27].

### 3.2. Antigen Presentation, T-Cell Activation, and B-T Cell Cooperation

B cells can act as efficient antigen presenting cells (APCs) to stimulate T cells and allow optimal development of memory in the CD4+ T-cell population. Compared with nonspecific uptake associated with professional APCs, selective uptake of antigen by antigen-specific B cells is markedly superior [28]. Furthermore, RF+ B cells, in particular, are believed to play an important role in antigen presentation [29]. They can take up antigen-Ig immune complexes via their membrane Ig receptors, which have RF specificity. B cells then process and present peptides from the antigen and thus induce both T-cell activation and T-cell help [29].

Evidences that T-cell responses in RA synovitis can be dependent on B cells come from studies by Takemura and coworkers [30]. Treatment with a monoclonal anti-CD20 antibody in severe combined immunodeficient (SCID) mice xenotransplated with RA synovial tissue enriched in B cells and GCs led to disruption of GCs, loss of follicular dendritic cell (FDC) networks, and impairment of T cell activation, with fall in the production of T cell-derived cytokines.

The recent demonstration that B cells reacting toward citrullinated peptides are enriched in RA joints [25], together with the well-recognized expression of citrullinated proteins in the same anatomic location [31], suggests the possibility that these mechanisms may also be involved in the amplification of autoimmune responses in human disease through a local cross-talk between citrullinated peptide-reacting B cells functioning as APC for citrullinated peptide-specific synovial T cells.

Besides antigen presentation, B cells could stimulate pathogenic T-cell responses through cytokine secretion. A candidate cytokine for such function is IL-6. B cells are a relevant source of IL-6 in the chronic phase of autoimmune diseases [32], including RA [33]. Mice deficient in IL-6 have reduced arthritis severity coupled with impaired IL-17 production by CD4+ T cells [34]. Furthermore, IL-6 inhibition increases the frequency of functionally suppressive Tregs in both experimental and human arthritis [35]. IL-6 has indeed been recently identified as a major regulator of the balance between effector T helper (Th)17 cells and Tregs. In the presence of transforming growth factor (TGF)-*β*, IL-6 induces de novo differentiation of Th17 from naïve T cells through upregulation of retinoid orphan receptor-*γ*t, while it inhibits TGF-*β*-induced Treg differentiation [36]. In turn, Th17 cells and Th17 cell derived cytokines have been shown to promote B-cell proliferation, differentiation, class-switch recombination, and antibody production in vivo [37], suggesting the existence of a positive feedback loop between T and B cells potentially involved in the amplification of the inflammatory response. In keeping with these concepts, response to treatment studies on humans demonstrate the effect of B-cell depleting agents on both B lymphocytes and Th17 responses in vitro [38]. Whether these mechanisms are part of chronic synovitis pathogenic cascade in vivo still remains to be proved.

### 3.3. Cytokine Production

Besides the potential contribution of B cells to T-cell differentiation and function, there is now growing evidence indicating that B cells in RA can directly contribute to the local synthesis of a wide spectrum of cytokines acting on other pathologically relevant cell types. Yeo et al. [33] through gene expression analyses of sorted cell populations have demonstrated a generally proinflammatory role of synovial fluid B cells with recognition of high expression of the subunits required for functional IL-12 and IL-23 production, as well as of IL-1*α* and tumor necrosis factor (TNF)-*α*. Of considerable relevance the same authors have shown that B cells represent a major source of receptor activator nuclear factor kappa B ligand (RANKL) in the rheumatoid environment, suggesting their direct involvement in the process of osteoclastogenesis. In keeping with these concepts, recent reports have also shown that CD19+ B cells from both RA patients and healthy individuals are capable of producing IL-17A [39], a pleiotropic cytokine involved in multiple aspects of immune function, inflammation, and bone damage. There is also evidence that B lineage cells infiltrating the inflamed synovium may induce cytokine synthesis in synovial fibroblasts through the production of IL-36*α* [40], suggesting the capacity of tissue infiltrating B cells to interact not only with haematopoietic cells but also with the local stroma through paracrine mechanisms.

### 3.4. Immune Regulation

Whilst the above paragraphs would support the role of B cells as humoral effectors, APCs, and proinflammatory cytokine producers, there is now evidence that specific subsets of B cells can actually function as regulatory elements, controlling local inflammatory pathology. Recent studies on humans demonstrate that CD19+CD24hiCD38hi peripheral blood B cells, a phenotype overlapping the one characteristic of recent bone marrow emigrant B cells (transitional B cells), can exert suppressive activity in vitro. In coculture systems, activated CD19+CD24hiCD38hi B cells can indeed suppress T-cell proliferation, inhibit naïve T-cell differentiation into Th1 and Th17 cells, and convert CD4+CD25− T cells into Tregs, partially via the provision of IL-10 [41–43]. Although several aspects of regulatory B-cell (Breg) pathobiologic involvement in RA remain to be clarified, there is now evidence that their suppressive function is partly impaired during active disease. The functional impairment of RA Bregs in vitro is coupled with their reduced frequency in the systemic circulation compared to healthy controls, a phenomenon detectable from the early phases of the disease [44] and possibly linked to their active recruitment to the inflamed joint [42]. These data suggest the potential localization of CD19+CD24hiCD38hi Bregs in the inflamed synovium and their participation to human synovitis dynamics. Additional studies are required to confirm these hypotheses to understand their functional relationship with other infiltrating lymphocytes and with synovial B-cell responses in situ.

### 3.5. Bone Homeostasis

Pathologic bone remodeling is a hallmark of RA. The skeletal complications of RA consist of focal erosion of marginal and subchondral bone, juxta-articular osteoporosis, and generalized bone loss. Although it is well established that the enhanced activity of osteoclasts results from the activation of T cells and the secretion of proinflammatory cytokines [45], a previously unappreciated role for B cells in regulating bone homeostasis has recently emerged. A step forward was made by the recognition that purified human autoantibodies recognizing citrullinated vimentin are able to promote the differentiation of mononuclear cells to osteoclasts in vitro and therefore directly promote bone loss [46]. Accordingly, ACPA-positive RA patients show more pronounced trabecular bone resorption at the distal radius compared to seronegative patients independent of disease duration, activity, and treatments [47]. Furthermore, high-resolution peripheral quantitative computed tomography of the metacarpal bones has revealed significant bone porosity in healthy individuals with ACPA compared with controls without immune reactivity to citrullinated proteins [48]. Whether ACPA are directly causative of bone resorption in human disease remains to be proven. An alternative or complementary hypothesis could be that immune responses against citrullinated proteins elicit subclinical levels of inflammation which in turn negatively affect bone homeostasis through conventional mechanisms. Supporting this view, some proinflammatory cytokines are upregulated in the serum of individuals later developing RA [49], and ACPA can acquire a proinflammatory Fc glycosylation phenotype before the onset of clinical disease [50].

Although less explored, B-cell pathology could be linked to bone remodeling also besides autoantibodies. As already discussed, B cells in the joints of patients with RA may directly participate to RANKL synthesis [33] and indirectly modulate the RANKL/osteoprotegerin (OPG) system through the production of proinflammatory cytokines, such as TNF and IL-17A [33, 39]. The net effect of B cells on bone homeostasis in RA remains however highly controversial and possibly depends on several factors including the developmental stage, the activation state, and the microenvironment in which B cells are embedded. Accordingly, B cells infiltrating the subchondral bone marrow of eroded joints in patients with long-standing disease may be rather involved in healing processes through the production of bone morphogenetic proteins [51]. In line with this observation, it has been recently shown that repair of bone erosions in RA patients treated with TNF-inhibitors, although rare, is based on bone apposition at the base of erosion and probably involves the bone marrow [52].

## 4. B Cells in Different Compartments

The analysis of B cell distribution, function, and alterations in the systemic circulation, the synovial tissue, and juxta-articular locations has been the object of several studies on RA and experimental models of arthritis, aiming at dissecting disease immune-dynamics as well as site-specific targets for biomarker and therapeutic development.

### 4.1. Peripheral Blood

Although assessments in the peripheral blood may present minor sensitivity if compared to inflamed target tissues, the circulation clearly represents an important anatomic district for pathogenic and translational studies, being representative of the systemic homeostasis and an easily accessible compartment for serial measurements in humans. In systemic diseases characterized by prominent autoantibody production and B-cell disturbances, such as systemic lupus erythematosus, several studies have indeed consistently shown differences of certain peripheral B cell subsets compared to healthy controls [5].

Data are not equally robust in RA, perhaps because of variability in disease phenotypes or pathobiology across different phases. Disturbances in the naïve B-cell compartment have been described in small cohorts of very early (<6 weeks of disease duration) and early (<6 months of disease duration) RA patients, with a slight (1–1.2 fold) increase in the frequency of IgD+CD27−CD19+ B cells compared to healthy controls [53, 54]. However, disease duration, therapy, or both seem to impact the circulating naïve B-cell pool, as no significant alterations or decreased frequencies are observed in patients with established disease treated with either synthetic or biological disease modifying antirheumatic drugs (DMARDs) [53, 55, 56].

In contrast, changes in the memory B-cell compartment appear more consistent, with most of the studies suggesting a reduction in the percentage of CD27+ memory B cells, involving either the IgD+ preswitched population [53–55] or the IgD− postswitched fraction [56] or both [57]. Memory B cells express the chemokine receptors CXCR5, CXCR4, and CCR7 [58] that account for their migration across different anatomic compartments under homeostatic and inflammatory conditions. Serum levels of their ligands CXCL13, CXCL12, and CCL19 (which may well reflect the degree of expression of these factors in peripheral tissues) have been actually shown to be inversely correlated with the frequency of blood memory CD27+ B in RA [59], suggesting that there might be a migration or redistribution of pathogenic memory B cells into different anatomic compartments, such as the inflamed synovium. In keeping with this concept, synovial B cells are enriched in CD27+ memory B cells compared to the peripheral blood [51, 55, 58].

### 4.2. Synovial Tissue

**Figure 2:**
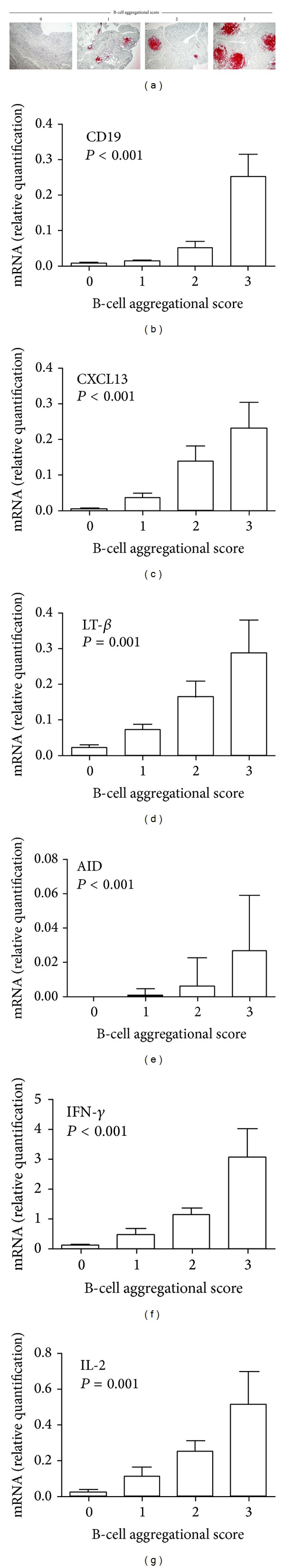
The gradient of B-cell infiltration and aggregation in rheumatoid synovitis. (a) Representative examples of progressive degrees of synovial B-cell infiltration in specimens from 4 independent patients with rheumatoid arthritis stained for the B-cell marker CD20 are shown. Original magnification: 100x. (b) Validity of the B-cell aggregational score is confirmed by parallel immunohistochemical and mRNA expression analysis for the B-cell marker CD19. ((c), (d)) The progressive enrichment in synovial B aggregates is coupled with progressively increasing levels of the B-cell chemoattractant CXCL13 (c) and the cytokine lymphotoxin (LT)-*β* (d), known to regulate lymphoid tissue ontogenesis and neogenesis. ((e)–(g)) B-cell aggregation is associated with the progressive increase in markers reflecting immune cell activation, such as the B-cell activation marker activation-induced cytidine deaminase (AID) (e) and the T cell-derived cytokines interferon (IFN)-*γ* (f) and interleukin (IL)-2 (g). The graphs show mean (SD) expression levels stratified according to the B-cell aggregational score.

Compared with other conventional cell populations, such as macrophages and T lymphocytes, B cells infiltrating the inflamed synovium in RA show the highest degree of qualitative and quantitative heterogeneity [60, 61]. A variable but yet considerable proportion of synovial tissues indeed completely lacks B cells. When present, B cells are virtually restricted to follicular structures with wide variation in size and density distribution (Figure 2). These structures are the preferential environment in which topographic interactions between B cells and T cells, macrophages, mesenchymal stromal cells, and dendritic cells are favoured, which makes them the elective intrasynovial site for potential intercellular (cell-contact dependent or paracrine) immunological interactions to take place [62]. In keeping with this concept, progressive enlargement of intratissue lymphoid structures and local B-cell enrichment can be associated with acquisition of molecular, cellular, and structural features constitutive of secondary lymphoid organs. These features include the constitution of partly separated B- and T-cell rich areas, local production of homeostatic lymphoid chemokines (CXCL13, CCL21), and differentiation of stromal cells typical of lymphoid tissues, involved in local antigen- and cell delivery in the B- and T-cell compartments (smooth muscle actin [SMA]+ CCL21+ CD45− fibroblastic reticular cells, CD21+ FDCs, peripheral node addressin [PNAd]+ high endothelial venules) [63–65]. Despite the acquisition of the above mentioned structural features is not systematic, with frequent observation of partly organized aggregates, there is molecular evidence indicating that the process of B-cell infiltration in RA can lead to functionally-competent immunological niches characterized by GC-like activity. Several in situ data appear to support this concept: (i) the recent demonstration of local expression of the enzyme AID and its association with local detection of circular transcripts, transient by-products of the ongoing process of immunoglobulin class-switch [66]; (ii) the analysis of B cell aggregates micro-dissected from synovial tissue samples showing somatic diversification of the V-gene repertoire [67]; (iii) V-region gene analysis of B cells and plasma cells from the same tissues suggesting that synovial plasma cells are largely generated from locally activated B cells [68].

The local activation of autoreactive B cells may be involved in the generation of pathogenic autoantibodies. As previously mentioned, monoclonal IgG antibodies generated from joint-derived B cells of RA patients have actually a strong bias toward citrullinated autoantigen recognition [25], and AID-expressing follicular units from RA synovium xenotransplanted into SCID mice are associated with circulating human IgG ACPA in mouse sera [66]. Accordingly, in human samples, AID expression levels are associated with IgG ACPA titres in ACPA-positive patients, though high AID synovial expression can be also observed in ACPA-negative patients, and, conversely, ACPA positivity may occur in the absence of significant B-cell infiltration in the synovium (S. Bugatti, unpublished observations).

As already mentioned, synovial B cells may orchestrate additional pathogenic processes besides autoantibody production, such as MHC class II-dependent T-cell activation [30]. Accordingly, tissues enriched in B-cell aggregates display the highest levels of the T cell-derived cytokines interferon (IFN)-*γ* and IL-2 (Figure 2).

### 4.3. Bone Marrow

In humans, the largest part of B-cell development occurs in the haematopoietic red marrow. Furthermore, plasma cells establish their survival niche in the bone marrow. The involvement of the systemic bone marrow as a primary site for autoreactive B-cell development and autoreactive plasma cell survival in RA is thus unsurprising. Less expected, under chronic inflammatory conditions including RA, the systemic bone marrow may acquire morphological features of a secondary lymphoid organ. In a unique series of 15 bone marrow trephine biopsy specimens from RA patients, 9 exhibited a follicular pattern of infiltrating lymphocytes, and GCs were detected in 5 [69]. Isolated reports have also described B-cell clonality occurring in association with marrow lymphoid follicles [70].

Evidence has accumulated over the years that bone marrow changes in RA also involve the subchondral bone marrow of peripheral joints [71]. This is normally a fat-rich tissue devoid of significant immunologic activity. In eroded joints of patients with long-standing RA, the fat is replaced by an immune-inflammatory infiltrate that organizes into follicles [51, 72, 73]. Although marrow follicles share most of the morphological features of synovial lymphoid aggregates [51, 72], they are enriched in memory B cells and plasma cells [51]. Functionality at these sites has yet to be demonstrated, and the actual contribution of subchondral bone marrow B cells to the immunological disturbances of RA is not proven. However, since the recognition that bone marrow involvement in magnetic resonance imaging (bone marrow edema) occurs very early in course of the disease [74, 75] and strongly predicts radiographic progression [76], it has been tempting to speculate that inflammation and lymphoid infiltration within the subchondral aspect of the joints in RA patients actively contribute to local bone damage. Though, as already mentioned, the effects of B cells on bone remodeling might be variable also depending on the anatomical sites in which their activation occurs. Accordingly, both factors involved in bone resorption and factor promoting new bone formation have been described at the subchondral bone marrow level [51, 73], and definitive conclusions on the functional significance of marrow follicles cannot be drawn from current knowledge.

### 4.4. Lymph Nodes

Secondary lymphoid organs are a primary site for the generation of adaptive responses and for the regulation of inflammatory reactions in peripheral drained tissues. Understanding the biological processes related to B cells in secondary lymphoid organs in course of autoimmunity is therefore a central issue with considerable scientific and clinical perspectives. The scarce accessibility of these compartments in humans through invasive procedures has however limited our comprehension of their behavior during RA, with most of the available data derived from studies on experimental animal models.

In this context, lymph node involvement appears as an early event and has been recognized as a fundamental component of the arthritis process. An increase in the percentage of B lymphocytes as well as a high proliferation of CD8+ cells was observed in regional lymph nodes in the latency period of adjuvant arthritis [77]. In the K/B × N model of spontaneous autoimmunity, the lymph nodes draining the distal joints were found essential for the amplification of the arthritogenic B-cell response [78]. Similarly, structural changes in lymph nodes have been reported in TNF-transgenic mice [79, 80], with evidence indicating a role of local B cells in different aspects of the disease. Lymph nodes draining inflamed joints are indeed selectively characterized by local differentiation of CD23+CD21highCD1dhigh B cells (a population defined “Bin”, B cells in inflamed nodes) [81, 82]. Such cell population has been shown to display an enhanced ability to capture and process antigen-immune complexes, to express greater levels of MHC class II and costimulatory antigens and to exhibit a GC phenotype at higher rate compared with follicular B cells [82]. These data suggest that draining lymph nodes can accumulate B cell subsets with enhanced immunological potential, which may contribute to autoimmunity progression in predisposed individuals. Of considerable relevance, B cells and Bin localized in draining lymph nodes from TNF-transgenic mice have been shown to play a role in arthritis progression also through nonimmunological mechanisms. B cells accumulating in reactive lymph nodes can indeed translocate from the follicular area to the sinusoidal lymphatic sinuses, impairing afferent lymphatic drainage and leading to lymph node collapse, a process associated with increased inflammation and erosive changes in the drained joint [83]. These events have been shown to be reversible by B-cell depleting agents, explaining a potential alternative mechanism of action of B cell-targeted therapies in RA [84].

Although the specific relevance of these findings to human disease is currently unknown, there is growing evidence indicating that draining lymph nodes can be integrating part of RA pathologic processes. Former histologic studies on lymph node biopsies from different anatomic sites in established RA described follicular hyperplasia and interfollicular plasmacytosis [85] as well as increased GCs with high B-cell activity [86]. More recent data exploiting power Doppler ultrasound demonstrated the possibility to capture significant structural and vascular flow changes in axillary lymph node of patients with active disease, including hypertrophy of cortical regions [87]. Supporting these data, flow cytometry analysis of inguinal lymph node cells isolated from ultrasound-guided biopsies in in early arthritis patients showed an increase in activated CD69+ CD8+ cells and CD19+ B cells compared to healthy controls, suggesting an increased immune cell activation within the lymph node compartment [88]. Ultrasound-guided biopsy of inguinal lymph nodes appears feasible and safe [89] and promises to yield important information in the next future.

## 5. B Cells as Biomarkers

The discovery of the pathophysiological role for B cells in regulating several aspects of the immune response in RA has greatly refined our understanding of the disease over the last decade. Despite common considerations reviewed so far, RA remains a heterogeneous syndrome in terms of clinical expression and long-term course, and different pathogenic pathways are likely to be differently activated in different patients or at least in different phases of the disease. In particular, the relative contribution of B lymphocytes appears greatly variable, as inferred at least by the existence of a seropositive and a seronegative subtype of RA. As a consequence, the B-cell signature is also being extensively investigated in its possible clinical applications, in search of new biomarkers able to complement and expand the well-established diagnostic and prognostic value of RA-related autoantibodies. The reader is referred to comprehensive reviews on the clinical significance of ACPA and RF in RA [90, 91]. Here, we will summarize recent data on non-antibody B-cell biomarkers.

### 5.1. Disease Diagnosis

#### 5.1.1. Serum B-Cell Markers and B Cell-Related Factors

The performance of markers of B-cell activation in the classification of early inflammatory arthritides has been extensively investigated in the ESPOIR cohort, a large French multicenter prospective cohort including patients with early arthritis of <6 months duration [92, 93]. In this cohort, serum markers of B-cell activation, such as levels of IgG, IgA, free light chains (FLC) of Ig and *β*2-microglobulin, have been reported to be increased in early RA compared to undifferentiated arthritis (UA) [92]. Furthermore, some of these markers, such as *β*2-microglobulin, maintained an independent predictive ability for RA diagnosis in multivariable regression models also including ACPA and acute phase reactants [92]. Importantly, neither the increase in BAFF serum levels nor BAFF gene polymorphism could discriminate early RA from UA [92]. However, caution should be put in interpreting the clinical associations of BAFF serum levels in RA and autoimmune diseases in general. In fact, BAFF family members exist in several different forms depending on protein structure, splice variants, glycosylation, and cleavage, each with possible specific biological activities [94], and BAFF quantification based on ELISA commercial kits was found to be largely inaccurate [95].

Further studies into the ESPOIR cohort have confirmed that, among a broad panel of cytokines investigated, IL-6 and IL-21 only were associated with increased proportions of autoantibodies and higher levels of markers of B-cell activation [93]. Again, baseline levels of IL-6 could discriminate RA from UA with a positive predictive value of 85.8% and a negative predictive value of 32.8% [93]. In multivariate analysis, the association of IL-6 with RA diagnosis was independent of ACPA positivity and markers of inflammation, with an OR of 1.9. Higher levels in RA compared to UA were also found for IL-21. It should be noted that a proportion of patients classified as suffering from UA at baseline also displayed high levels of markers of B-cell activation [92]. In this context, novel biomarkers reflecting RA pathogenesis could be tested for their performance in improving the recognition and management of chronic arthritides since the very early phases of the disease, before the fulfillment of any established classification criterion [96].

#### 5.1.2. Synovial Tissue B Cells and B Cell-Related Factors

In 1978, Goldenberg and Cohen [97], after histopathologic evaluation of synovial tissues obtained from 90 patients suffering from different rheumatic diseases, concluded that the presence of lymphoid follicles could be regarded as a specific feature of RA. Over the years, several investigators have revised this assumption demonstrating that most of the chronic inflammatory arthritides share common histopathologic features, including the local organization of infiltrating mononuclear cells into aggregational structures [98–100]. A recent prospective study involving 93 patients with early arthritis of <12 months duration has failed to demonstrate any diagnostic value of synovial lymphocyte aggregates evaluated by means of T-cell markers [100]. Although the wide heterogeneity in synovial tissue patterns observed among patients with RA and the high degree of overlapping among different disease entities make the use of synovial tissue analysis at present unrealistic for diagnostic purposes, caution should be put in interpreting the current controversies in literature. No standard consensus indeed exists on the classification of synovitides in either diffuse or aggregational, as the two patterns of infiltration actually represent a continuum of overlapping forms of variable intensity rather than mutually exclusive histotypes [60]. Accordingly, preliminary data in 84 sequentially recruited patients with at least 1 swollen joint and disease duration <12 months indicate that the presence of synovial aggregates does associate with baseline RA diagnosis according to the 1987 criteria [101], thus reopening the dispute on the clinical significance of lymphocyte infiltration patterns. An additional layer of complexity resides on the histological criteria adopted to identify lymphocytic aggregates (B cells only/T cells only/B + T cells). When specifically looking at B cells as core elements of lymphoid clusters, the numbers of CD38+ plasma cells and CD22+ B cells (but not of CD3+ T cell aggregates) were the best discriminating markers comparing RA to non-RA in 95 patients with active UA at time of presentation [102].

### 5.2. Disease Phenotype and Prognosis

#### 5.2.1. Serum B-Cell Markers and B Cell-Related Factors

Overall, markers of B-cell activation in early RA appear mostly associated with autoantibodies as well as with inflammatory features [92]. The interpretation of their possible prognostic value thus requires caution and should take into account confounding factors. Yet, in the ESPOIR cohort, some B-cell markers, such as total IgA and kappa FLCs, turned out to be independently associated with radiographic erosions at disease onset [92], and IL-6 and IL-21 serum levels were also predictive of rapid radiographic progression at 1 year irrespective of clinical inflammation [93].

Studies analyzing the prognostic value of the B-cell chemoattractant CXCL13 are opening promising perspectives in the field of biomarker discovery in RA. CXCL13 is critically involved in several autoimmune diseases by redistributing B lymphocytes into injured tissues, organizing their microanatomical positioning and possibly enhancing their BCR-mediated activation [103]. Serum levels of CXCL13 are increased in early RA patients compared to healthy controls [104, 105], and synovial CXCL13 has been shown to correlate with accumulation of CXCL13 protein in the serum [106, 107]. CXCL13 appears as a marker of severity in RA. A large prospective study has indeed shown that early RA patients with the highest levels of serum CXCL13 are those with the highest rate of progression of joint damage over long-term follow-up [104]. In line with these data, in 161 untreated RA patients with disease duration <12 months, baseline CXCL13 levels >100 pg/mL predicted persistent subclinical ultrasonographic synovitis despite effective treatment with conventional DMARDs [105]. Although CXCL13 levels at baseline correlated with the ACPA status as well as with disease activity, the predictive value of the chemokine appeared independent of both factors [104, 105]. Important issues that need to be clarified for serum CXCL13 to be established as an additional biomarker include demonstration of whether circulating levels of the chemokine reliably reflect synovial synthesis and different synovial pathotypes and how well they outperform currently available tests in the clinical assessment and prognostic stratification of RA.

#### 5.2.2. Synovial Tissue B Cells and B Cell-Related Factors

Because RA synovia harvested from different RA patients show wide variations in the size and density distribution of B-cell aggregates, a question which therefore arises is whether such heterogeneity translates into specific phenotypic differences and variable disease outcomes.

In animal models, inflammatory stimuli promote the local migration and retention of B lymphocytes within nonlymphoid tissues through the induction of chemoattracting factors, such as CXCL13, but they are no further required for their maintenance [108, 109]. Accordingly, synovial levels of CXCL13 expression and lymphocyte infiltration appear associated with features of local disease activity in early RA [100, 107], whilst the correlation is weaker or even lost for longer duration [106, 107, 110]. Thus, “B-cell synovitis” does not per se appear synonymous to clinically active disease. Despite the lack of overt inflammatory features, continuous synovial expression of CXCL13 may mark ongoing immune cell activation and the establishment of a local milieu favouring tissue remodeling, as suggested by increased levels of AID, IFN-*γ*, and IL-2 expression and a higher RANKL/OPG ratio in tissues expressing increasing levels of the chemokine [107]. Accordingly, patients displaying the highest levels of CXCL13 appear to suffer from more severe RA in cross-sectional evaluations [107]. Similarly, the density of CD79a-positive B cells has been shown to positively correlate with radiographic joint damage, which was instead unrelated to other inflammatory features such as T cells, lining or sublining macrophages, and capillary angiogenesis [111]. Whether such cross-sectional association also has prognostic significance over long-term follow-up has however remained difficult to establish, and large prospective studies on early RA are currently not available. Yet, in a small cohort of 18 patients with short disease duration, the degree of synovial CD20 expression at the metacarpophalangeal joints was shown to significantly correlate with radiological damage at 3 years independent of other features of local inflammation [112]. Overall, the few available studies seem to suggest that local B cell-centred processes might somehow be associated with features of disease severity. Importantly, these could be eventually captured prospectively if the B-cell pathotype of synovial inflammation is analyzed in its quantitative and functional features. Given the strong relationship between synovitis and bone damage, the exploration of possible biomarkers of disease severity in this area could be conceivably fruitful [113].

### 5.3. Response to Therapy

#### 5.3.1. Peripheral Blood B Cells, B-Cell Markers, and B Cell-Related Factors

Given the availability of therapies directly targeting B cells, research on the possible role of B-cell disturbances in predicting response to treatment has naturally focused on rituximab.

Rituximab induces nearly complete depletion of CD20+ B lymphocytes in the peripheral blood which is sustained for several months after treatment [114]. Reconstitution of peripheral blood B cells first involves immature B cells, followed by naive B cells. In contrast, memory B cells show a slow and delayed repopulation [115–117]. Interestingly, better response to rituximab and late relapse in responders have been associated with decrease in memory B cells [56, 116, 117], confirming the pathogenic involvement of this cell population in RA. Furthermore, a higher number of pre-plasma cells before treatment have been associated with incomplete B-cell depletion and a worse clinical response [118], and elegant studies analyzing quantitative mRNA assays for B lineage cells have confirmed that markers for antibody-secreting plasmablasts predict nonresponse to anti-CD20 therapy [119].

Because extensive phenotyping of B-cell subpopulations is costly and not easily accessible in routine care, predictors of response have been also searched within standard markers of B-cell activation, such as serum Igs, FLC, and BAFF. In the SMART study, involving 208 patients with active RA in whom anti-TNF agents had failed or were contraindicated, serum IgG > 12.66 gm/liter (upper limit of normal) arose as independent predictors of response to rituximab with an OR of 2.11 and synergistic with RF and/or ACPA [120], confirming that anti-CD20 treatments may be particularly indicated in the B cell-driven subtype of RA. Although BAFF levels were not found to be associated with clinical response, a recent study analyzing 138 seropositive RA patients indicates BAFF levels >1011 pg/ml as an independent predictor of a good EULAR response in this subgroup of patients [121]. Again, contrasting results might depend on the different clinical features of the patients' population being analyzed, as well as on the inaccuracy of BAFF serum level measurements due to the biological complexity of this cytokine [94, 95].

Since trafficking and repopulation patterns of B cells following rituximab are relevant to the mechanism of action of rituximab itself, recent studies have focused on the role of B cell attracting chemokines as potential markers of response. In a small open label study of rituximab involving 20 patients with active RA (ARISE trial), high baseline serum levels of CXCL13 were predictive of B-cell repopulation at 6 months after treatment [106]. Although in the larger SMART study CXCL13 was not associated with EULAR response at week 24, another chemokine involved in B-cell migration, that is, CCL19, turned out to be predictive in a multivariable model not including autoantibodies [59].

#### 5.3.2. Synovial Tissue B Cells and B Cell-Related Factors

Whilst variations in the degree of sublining macrophage infiltration can early and reliably track the clinical efficacy of conventional and experimental treatments in RA [122], the effects of therapies on synovial B cells/B-cell markers and the ability of B-cell infiltration to predict different clinical responses are less clear.

Although not directly addressed, conventional DMARDs do not seem to significantly affect synovial B-cell pathways. A recent study comparing the effects of tocilizumab with methotrexate failed to report significant changes in B cell and plasma cell numbers in 15 patients with early RA receiving standard dosages of MTX for 6 months [123]. Relevantly, gene expression levels of CXCL13 also remained unchanged [123], confirming that this chemokine may be a marker of more refractory synovitides with worst disease evolution upon conventional treatments [104–107]. In contrast to synthetic DMARDs, biological drugs may interfere with local B-cell pathology. Reductions in B-cell numbers or loss of follicular architecture have been reported in association with effective treatment with either TNF-inhibitors, tocilizumab, or abatacept [110, 123–125]. Also, the presence of well-organized B-T cell follicles has been proposed as an independent predictor of lower response to anti-TNF agents [110].

Synovial tissue analysis following rituximab treatment has provided suggestive results demonstrating that, compared to the almost complete depletion of CD20+ B lymphocytes in the peripheral blood, depletion in the synovium is less complete and varies between patients irrespective of the clinical response [126–129]. In 24 RA patients undergoing serial synovial biopsies, B-cell levels at baseline were not predictors of response. Clinical response was instead predicted by the reduction of plasma cells, presumably as a consequence of depletion of their memory B cell precursors [127]. Accordingly, a low disease activity state following rituximab was associated with reduced infiltration of CD79a+ CD20− plasma cells in the synovium [129]. Collectively, these findings support the notion that therapies aimed at interfering with B-cell pathology in RA could be improved through the targeting of additional players, such as CD20-negative plasma cells, as well as additional compartments, such as the synovial tissue.

## 6. Conclusions

The contribution of B lymphocytes to RA pathogenesis goes well beyond autoantibody production and extends to areas unsuspected until recent years. Knowledge will expand further with the entry of new B cell-targeted therapies into the market. The redistribution of B-cell subsets in different anatomic compartments during the course of the disease should be taken into account to dissect the intricate mechanisms of autoreactive B-cell activation and survival. Finally, and perhaps more relevantly, a broader insight into B-cell pathologic reactions could help generating novel biomarkers of disease diagnosis, prognosis, and response to therapy in patients with RA.

## Figures and Tables

**Table 1:** Autoantibodies described in rheumatoid arthritis.

Rheumatoid factors	
Anti-collagen type II	
Anti-glucose-6-phosphate isomerase (GPI)	
Anti-human cartilage glycoprotein 39	
Anti-Ra33/heterogeneous nuclear ribonucleoprotein (hnRNP) A2	
Anti-citrullinated fibrinogen	
Anti-citrullinated vimentin	
Anti-citrullinated alpha-enolase	
Anti-immunoglobulin binding protein (BiP)	
Anti-carbamylated proteins (anti-CarP)	
Anti-peptidyl arginine deiminase (PAD)	
Anti-histones	
Anti-*Porphyromonas gingivalis*-derived enolase	
Anti-***Porphyromonas gingivalis***-derived PAD	
